# Characterization of a rat osteotomy model with impaired healing

**DOI:** 10.1186/1471-2474-9-135

**Published:** 2008-10-08

**Authors:** Christine Kratzel, Camilla Bergmann, Georg Duda, Stefan Greiner, Gerhard Schmidmaier, Britt Wildemann

**Affiliations:** 1 Julius Wolff Institut, BCRT, CMSC Charité-Universitätsmedizin Berlin Augustenburger Platz 1, 13353 Berlin, Germany

## Abstract

**Background:**

Delayed union or nonunion are frequent and feared complications in fracture treatment. Animal models of impaired bone healing are rare. Moreover, specific descriptions are limited although understanding of the biological course of pathogenesis of fracture nonunion is essential for therapeutic approaches.

**Methods:**

A rat tibial osteotomy model with subsequent intramedullary stabilization was performed. The healing progress of the osteotomy model was compared to a previously described closed fracture model. Histological analyses, biomechanical testing and radiological screening were undertaken during the observation period of 84 days (d) to verify the status of the healing process. In this context, particular attention was paid to a comparison of bone slices by histological and immunohistological (IHC) methods at early points in time, *i.e*. at 5 and 10 d post bone defect.

**Results:**

In contrast to the closed fracture technique osteotomy led to delayed union or nonunion until 84 d post intervention. The dimensions of whole reactive callus and the amounts of vessels in defined regions of the callus differed significantly between osteotomized and fractured animals at 10 d post surgery. A lower fraction of newly formed bone and cartilaginous tissue was obvious during this period in osteotomized animals and more inflammatory cells were observed in the callus. Newly formed bone tissue accumulated slowly on the anterior tibial side with both techniques. New formation of reparative cartilage was obviously inhibited on the anterior side, the surgical approach side, in osteotomized animals only.

**Conclusion:**

Tibial osteotomy with intramedullary stabilisation in rats leads to pronounced delayed union and nonunion until 84 d post intervention. The early onset of this delay can already be detected histologically within 10 d post surgery. Moreover, the osteotomy technique is associated with cellular and vascular signs of persistent inflammation within the first 10 d after bone defect and may be a contributory factor to impaired healing. The model would be excellent to test agents to promote fracture healing.

## Background

Ten percent of all fractures require further surgical procedures because of impaired healing [1]. Coles [2] reported up to 17 percent of nonunions after treatment of closed tibial shaft fractures. Impairment of fracture healing is linked to demographic changes in society *e.g*. the growing proportion of elderly people and causes not only individual but also economic damage [3]. Hence, by means of *in vitro *and *in vivo *experiments, considerable efforts have been made in fracture research to develop therapeutic approaches. Before promising concepts can be used in humans, it is necessary to verify their efficacy and safety in a variety of animal models (for critical review and guidelines see [4]).

Models simulating impaired fracture healing in animals are not easy to conduct. Animal models of bony nonunion mainly utilize techniques with large segmental defects, thermic treatment of the defect region, instable fixation or combinations of these procedures [5-8].

Descriptions or comparative studies of the different methods used in fracture research are often limited in detail. However, understanding of the biological course of healing is essential for a therapeutic approach and the choice of an adequate animal model can be crucial for the experimental results. Furthermore, with respect to laboratory animal stress and avoiding recurrent failure, we believe that critical reports on an *in vivo *approach are essential.

The bone healing process is a special form of wound healing. According to Cruess and Simmons [9,10] the regular course of fracture healing can be broken down into different phases: i the reactive phase, including fracture, haematoma and inflammation whereby the initial inflammation is regarded as an activator of fracture healing [11]; ii the reparative phase, characterized by callus formation and lamellar bone deposition, and iii the remodeling phase, creating the original bone contour. During this process two types of bone formation are possible: *desmal *with direct bone formation from mesenchymal progenitor cells and *chondral *with bone formation from cartilage intermediate.

Impairment of regular osseous healing can result in delayed union or nonunion (also called pseudarthroses). Classification of these cases in humans depends on whether nonconsolidation of bone fragments occurs within 4 or 6 months [12,13]. Nonunions can be classified as hypertrophic and are termed as vital on the basis of radiological evidence of proliferative external callus formation on the fragment side; if callus formation does not occur, they are termed atrophic [14,15]. Common reasons for the impaired healing are poor end-to-end contact of the bone fragments, excessive interfragmentary movement and poor blood supply due to insufficient vascularization at the site of the defect or the interaction of all factors [16,17]. Infection is mentioned as another frequent cause of nonunion [18].

Rats are the most widely used animal species in bone healing research and osteotomy and closed fracture of long bones are frequently used model techniques [19]. Here we describe the course of delayed healing in an open rat tibial osteotomy model in comparison to a closed fracture model with regular healing in order to find a possible explanation for the impairment of healing.

## Methods

### Animal model

The animal experiments complied with German legal regulations and were approved by the responsible authorities. Adult female Sprague-Dawley rats (Harlan-Winkelmann) weighing 250–280 g were anaesthetized with isoflurane and by an intraperitoneal injection of a ketamine/xylazine mixture (80 and 12 mg/kg body weight, respectively). After shaving and disinfection of the right lower leg, the medullary cavity of the tibia was opened at the level of the proximal metaphysis and prepared for stabilization using a 1 mm Kirschner steel wire. The steel wire was removed and osteotomy was performed at the midshaft level of tibia using a diamond disk (Horico). The fibula was fractured manually. Osteotomy was stabilized intramedullary with a titanium Kirschner wire coated with PDLLA [20,21], a prospective carrier for therapeutics. After stabilization the wound was closed by a vicryl suture and gentamycin ointment was applied locally. For pain prophylaxis the animals received buprenorhine (0.05 mg/kg body weight s.c.) for the first 3 days after the intervention. Post mortem, in selected animals that underwent osteotomy, perifractural and titanium wire swabs (MWE) were taken for bacteriological examination. Bacterial growth was analyzed after 48 h of incubation in an external microbiological laboratory. The swabs were screened for anaerobic and aerobic bacteria.

The closed fracture of the tibia and fibula was produced in a standardized manner as published previously [21-23] and the stabilization of the fracture was performed in the same manner as described for the osteotomy model with a PDLLA-coated intramedullary nail.

Animals were regularly monitored radiographically. Mediolateral and anterior-posterior radiographs were taken postoperatively and at 28 d, 42 d and 84 d after surgery.

The animals were sacrificed under anaesthesia by intracardiac injection of calium chloride.

### Biomechanics

For biomechanical testing ipsilateral and contralateral tibiae of osteotomized animals (n = 18) killed at 28 d, 42 d and 84 d were dissected. The titanium wire was carefully removed under protection of the callus. Proximal and distal of the bone defect the tibia was Technovit-embedded into two moulds (Technovit 9100, Heraeus). Embedding of the contralateral tibia was performed analogous. Each mould was connected to a pivoted axis and the sample was preloaded with an axial force of 5 Newton. A constant linear propulsion (2 mm/min), generated by a material testing machine (Zwick 1455), was applied to a lever attached to one of the pivoted axis for transforming the translation of the material-testing machine to a uniform torsional movement. The other side was connected with a strain gauge (F_max _= 50 N, HBM) which recorded the torsional moment. Subsequently maximum load, torsional stiffness, and energy absorption were calculated. The data were expressed as percentages of those observed with the contralateral tibia not only to avoid interindividual differences in bone constitution, but also with respect to the curved anatomy and the varying cross section shape of the tibia at different levels. Biomechanical data of n = 25 animals, which had undergone the fracture technique [23], were compared to the open osteotomy model.

### Histology and histomorphometry

At days 28, 42 and 84 after osteotomy (n = 6 each time point) tibiae were carefully removed under protection of the callus bone and fixed for 2 days in 10% buffered formaldehyde and dehydrated in ascending concentrations of ethanol and then embedded undecalcified in methylmethacrylate (Technovit 9100, Heraeus). Longitudinal 6 μm thick sections were performed with a Leica SM 2500s microtome and were stained using a combination of Safranin-O/von Kossa for microscopic visualization of mineralized bone matter. Acrylate-embedded bone preparations from earlier studies using the fracture device [24] were used for comparison.

At days 5 and 10 bone specimen preparations were fixed in formalin for 2–3 days, decalcified with EDTA, processed in ethanol and xylene and embedded in paraffin. Longitudinal 6 μm thick sections were prepared and histological standard staining was performed with Haematoxylin-Eosin (HE), Alcian blue [25] and Movat-Pentachrom [26].

HE-staining was used to quantify the dimensions of reactive callus, Alcian blue staining to measure the cartilaginous fraction in callus (zones of hypertrophic chondrocytes) and Movat-Pentachrom to stain for the purposes of visualizing the fraction of newly formed trabecular bone in callus.

The dimensions of specific regions were measured in micrographs of adjacent slices using the Axio-vision 4.0 software. With respect to interindividual varieties, a region of interest (ROI) was established for each preparation *i.e*. the zone of reactive callus distally and proximally from the centre of bone defect which extended in length 1.5 fold the individual diameter of the cortical bone.

For immunohistological staining of vessels, the slices were rehydrated, blocked with normal horse serum and incubated over night with a monoclonal antibody against α-smooth-muscle actine (α-sma, Daco, dilution 1:100). A rat-absorbed anti-mouse biotin-conjugated antibody (diluted 1:50, 30 min incubation time at room temperature, Vector) served as the secondary antibody. The avidin-biotin-complex detection system (ABC method), coupled with alkaline phosphatase and Vector^® ^red as the chromogen, visualized the antibody binding (10 min incubation time at room temperature). The slices were counterstained with Mayers haemalaun solution (diluted 1:2). The morphology and amount of vessels in the callus were detected using an image analysis system (Zeiss KS 400). Slices from n = 6 animals each were evaluated 5 and 10 d post intervention (for osteotomy and fracture technique).

### Statistics

Biomechanical data of osteotomized and fractured tibiae (at 42 d and 84 d), dimension of whole callus, zones of reparative trabecular bone and zones of hypertrophic chondrocytes of ROI in both groups (at 5 d and 10 d) were compared using the Mann-Whitney U-test.

## Results

### Surgical procedure and monitoring

The surgical intervention of osteotomy was tolerated by all animals. Up to 3 days after surgery they received pain prophylaxis. However, one undesirable effect observed was that some animals licked their wounds inducing dermal lesions. In two animals there was a slight cranial shift in intramedullary nails.

**Figure 1:**
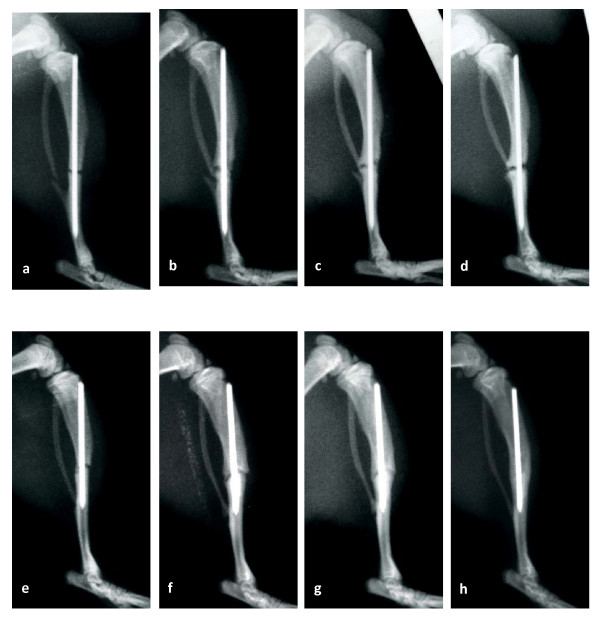
**(a-h)**. Radiographs of a rat tibia after osteotomy (a-d) or fracture (e-h) over the complete observation period: (a and e) day of surgery, (b and f) 28 d post surgery, (c and g) 42 d post surgery and (d and h) 84 d post surgery. Although there was the best possible fixation and there were no signs of osteolysis, radiographs of osteotomized animal revealed extremely impaired healing and clearly indicated nonunion.

In contrast to the fracture model [23] radiological screening of osteotomized animals revealed neither complete consolidation nor original remodelling of cortices until 84 d after surgery (Fig. 1a–h). The majority of animals showed hypertrophic bone fragments. In a minority of animals osteolytic zones could be observed.

### Biomechanics

Biomechanical testing of the tibiae of animals which underwent osteotomy revealed relatively low values for maximal torsional load and stiffness during the observation period (Table 1). In none of the animals did torsional load or stiffness reach comparable values to those recorded for the contralateral tibia. In contrast, the recently collected equivalent data from animals undergoing closed tibial fracture [[23], Pauly *et al*. unpublished data] revealed statistical differences against the osteotomy model with p = 0.0007 for 42 d and p = 0.0016 for 84d for both torsional load and stiffness, respectively. With respect to the small sample size in the osteotomized group at 28 d, due to unforeseen difficulties and damage when preparing the unstable tibial bone for measurement, the calculation of significances was not applied at this time point. However, the mean values in this group were clearly lower (Table 1).

**Table 1:** Biomechanical characterization of the osteotomized tibiae (torsional load and stiffness, as % of contralateral intact tibia, mean ± SD) compared to previously collected data for the fracture model.

Treatment	Torsional load	Stiffness	n =
Osteotomy 28d	15.1 ± 6.0	11.1 ± 9.1	3

Fracture 28d	69.0 ± 26.0	61.1 ± 44.5	9

Osteotomy 42d	29.3 ± 21.8	19.0 ± 15.8	6

Fracture 42d	129.3 ± 42.6 *	105.1 ± 38.6 *	8

Osteotomy 84d	48.0 ± 20.6	22.7 ± 12.9	5

Fracture 84d	173.9 ± 64.0 *	153.2 ± 53.8 *	8

Asterisks indicate significant differences versus the respective osteotomy groups. Due to the small sample size of osteotomized animals the statistical test for 28 d was not performed.

### Histological examinations

#### Screening of the healing process during the observation period (Safranin O/von Kossa staining)

**Figure 2:**
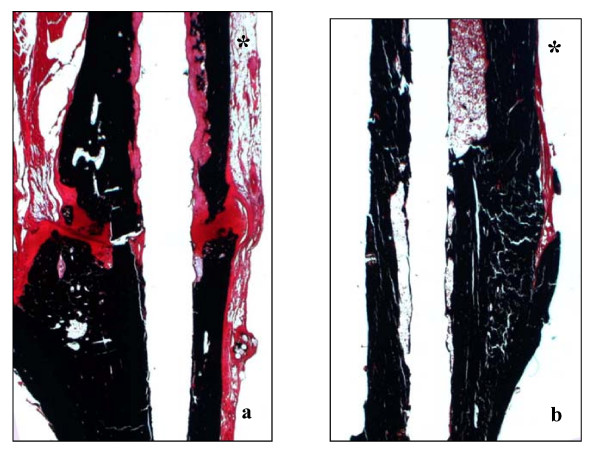
**(a and b)**. Safranin O/von Kossa staining to detect mineralized bone (black staining) of comparable areas in representative tibiae 84 d after osteotomy (a) and fracture (b) and intramedullary stabilization demonstrating nonunion and regular healing respectively. Cavities correspond to the 1 mm wire diameter. Asterisks indicate the proximal and anterior tibial side.

Histological screening at 28, 42 and 84d post surgery revealed microscopically a nonunion of osteotomized tibiae (Fig. 2a) except in 3 out of 6 examined animals at 84 d. These bone samples revealed incomplete union whereby only the periosteal callus was bridged. Histological slices of fractured tibiae [24] revealed bony bridging and status of remodelling until 84 d (Fig. 2b).

#### Early reactive callus, dimensions and cellular compartments (HE-staining)

**Figure 3:**
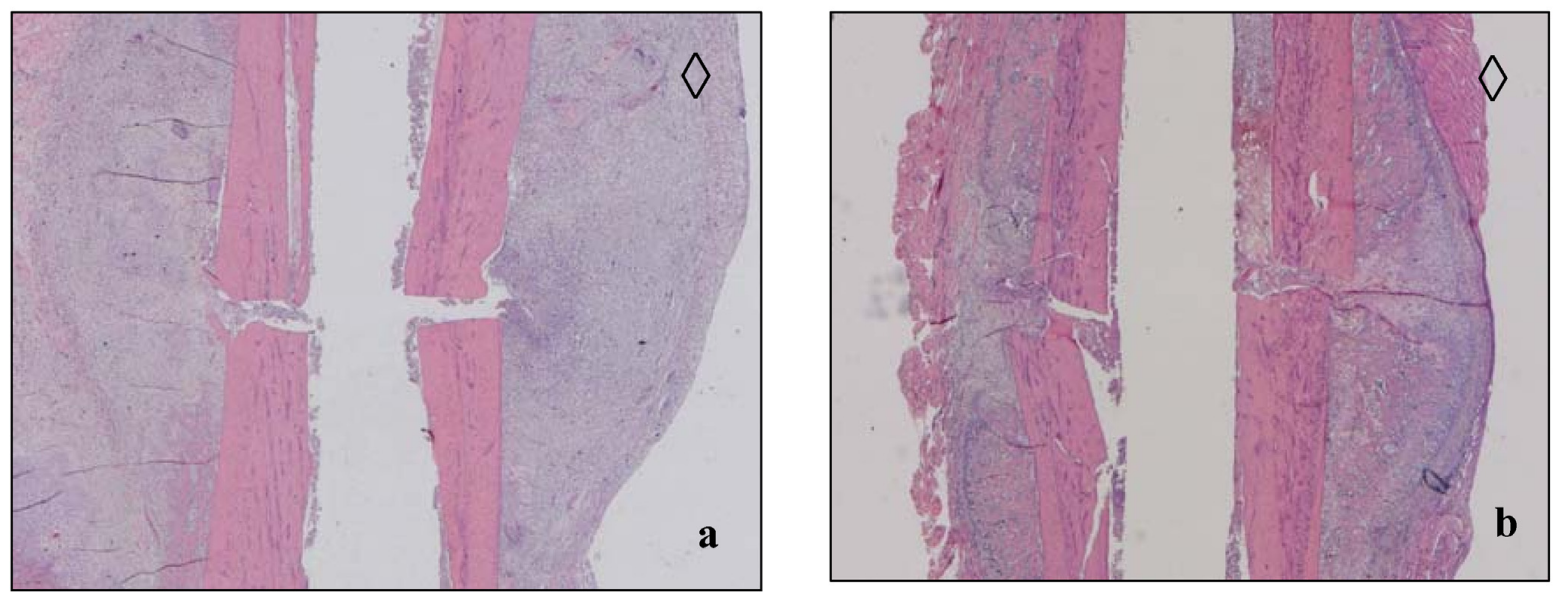
**(a and b)**. HE stained slices of comparable osteotomized (a) and fractured tibia areas (b) 10 d after surgery. The anterior tibial side is located at the bottom of pictures; diamonds indicate the anterior tibial side. Cavities correspond to the 1 mm wire diameter.

Dimensions of the reactive periostal callus were comparable at 5 d post osteotomy and fracture, respectively. However, 10 d after intervention the callus formation of the osteotomized animals was elevated compared to the fractured group (Figs. 3a and 3b; Table 2).

**Table 2:** Dimension of reactive callus (mm^2^) along the tibiae of osteotomized rats compared to fractured animals 5 and 10 days post surgery (mean ± SD).

Dimension in	whole callus	callus anterior	callus posterior
Osteotomy 5d	16.7 ± 4.6	13.2 ± 4.7	3.6 ± 0.7

Fracture 5d	12.6 ± 1.7	10.3 ± 1.7	4.1 ± 1.4

Osteotomy 10d	27.1 ± 11.3	15.7 ± 8.3	11.4 ± 4.4

Fracture 10d	16.0 ± 3.4 *	8.9 ± 2.3	7.0 ± 2.6

Asterisk indicates a significant difference versus the respective osteotomy group.

The cellular composition of callus consisted mainly of reparative granular cells (fibroblasts), inflammatory cells, chondrocytes and osteocytes or their progenitors and of extracellular matrix in both groups.

**Figure 4:**
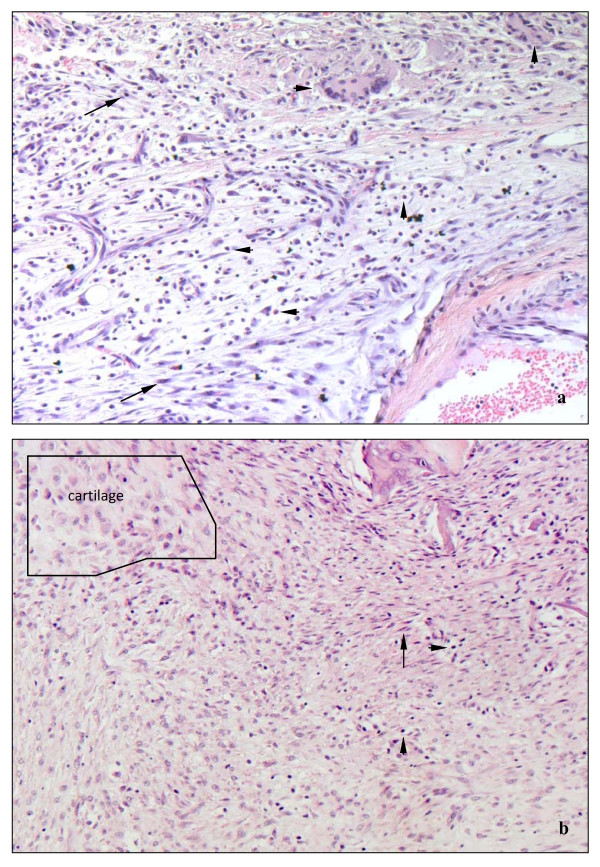
**(a and b)**. Micrographs of representative cellular types in callus adjacent to the bone defect after 10 d. Apart from fibrozytes and fibroblasts (arrows), osteotomized animals accumulate large numbers of lymphocytes, granulocytes and multinucleated giant cells (arrowheads). In contrast, callus in fractured animals hardly contained any inflammatory cells (b). HE staining, magnification 200×.

In osteotomized animals the appearance of inflammatory cells (neutrophils, lymphocytes, multinuclear giant cells) was clearly higher (Figs. 4a and 4b).

#### Early angiogenesis (a-sma staining)

**Figure 5:**
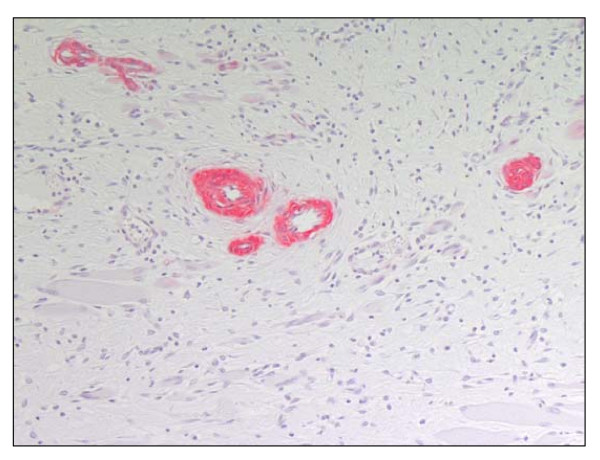
**Immunohistological staining of α-smooth muscle actin for selective staining of blood vessels (red labeled) in the callus of an osteotomized animal.** Magnification 200×.

Counting of blood vessels in a defined region of callus revealed similar vessel density at 5 d post operation in both groups, but significantly higher vessel density in osteotomized animals at 10 d post bone defect (p = 0.0043). This difference was more pronounced when comparing the anterior tibial sides (p = 0.0022, Fig. 5 and Table 3). Almost all of the counted vessels had a luminal diameter of approximately 20 μm.

**Table 3:** Amount of vessels per mm^2 ^in the callus (ROI) of osteotomized rats compared to fractured animals (mean ± SD).

Amount in	whole callus	callus anterior	callus posterior
Osteotomy 5d	46.6 ± 11.4	46.2 ± 18.4	39.6 ± 23.6

Fracture 5d	43.6 ± 15.0	50.2 ± 22.8	35.6 ± 5.9

Osteotomy 10d	37.0 ± 15.6	57.7 ± 25.2	14.6 ± 6.8

Fracture 10d	15.0 ± 4.8 *	18.2 ± 4.1 *	11.5 ± 7.2

Asterisks indicate significant differences versus the respective osteotomy groups.

#### Early fraction of woven bone and cartilaginous tissue (Movat Pentachrom staining and Alcian blue staining)

**Figure 6:**
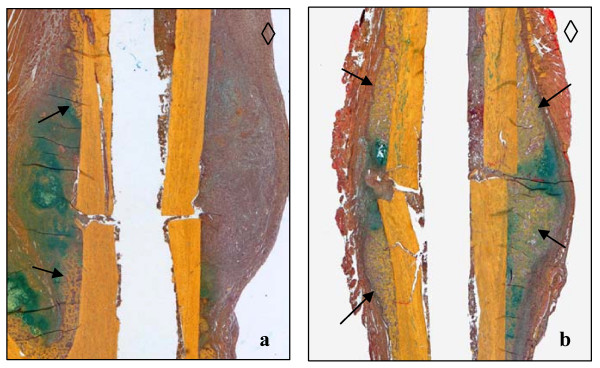
**(a and b)**. Histological preparation of comparable osteotomized (a) and fractured (b) tibial areas 10 days after surgery. Movat Pentachrome staining for detection of newly formed trabecular bone (arrows). Cavities correspond to the 1 mm wire diameter. Diamonds indicate the proximal and anterior tibial side.

**Figure 7:**
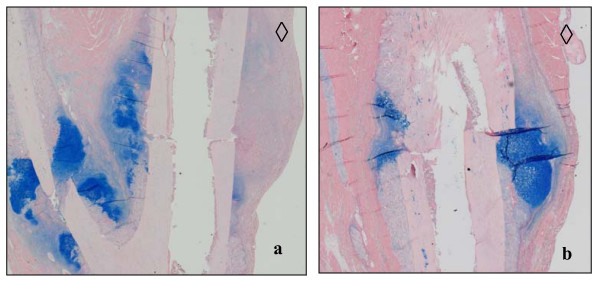
**(a and b)**. Histological preparation of an osteotomized (a) and fractured (b) tibia 10 days after surgery. Alcian blue staining for cartilage to detect hyperblastic zones of chondrocytes. Diamonds indicate the proximal and anterior tibial side. Note the homogeneous pattern of cartilage around the fractured fibula compared to the osteotomized tibia in the same animal (a). Cavities correspond to the 1 mm wire diameter.

Significantly more newly arranged bone was found in fractured animals at 10 d post operation than in the osteomized group (p = 0.0087). Moreover, in nearly all individuals in both groups, lower amounts of this tissue were observed on the anterior tibial side. This was not only the side with a minor soft tissue envelope but also the side of surgery (Figs. 6a and 6b; Table 4). More newly formed cartilage was found in the fractured animals 5 and 10 d post surgery (p = 0.026). Only osteotomized animals accumulated lower amounts of differentiated cartilaginous cells on the anterior tibial side (Figs. 7a and 7b; Table 5).

**Table 4:** Formation of newly formed bone in % of reactive callus (ROI) in osteomized rats compared to fractured animals 5 and 10 days post surgery (mean ± SD).

Amount in	whole callus	callus anterior	callus posterior
Osteotomy 5d	7.2 ± 9.6	8.2 ± 14.5	8.7 ± 9.8

Fracture 5d	8.9 ± 7.2	4.6 ± 5.7	16.4 ± 11.3

Osteotomy 10d	17.2 ± 8.4	7.5 ± 7.3	26.2 ± 9.1

Fracture 10d	34.8 ± 8.0 *	24.9 ± 7.8 *	48.8 ± 16.2 *

Asterisks indicate significant differences versus the respective osteotomy groups.

**Table 5:** Formation of cartilaginous tissue in % of the callus (ROI) in osteomized rats compared to fractured animals 5 and 10 days post surgery (mean ± SD).

Formation in	whole callus	callus anterior	callus posterior
Osteotomy 5d	0.2 ± 0.3	0.3 ± 0.5	0

Fracture 5d	2.25 ± 1.4	2.4 ± 2.2	1.9 ± 2.0

Osteotomy 10d	9.9 ± 7.7	2.8 ± 2.9	16.7 ± 13.7

Fracture 10d	22.1 ± 8.0 *	25.0 ± 9.0 *	17.6 ± 9.8

Asterisks indicate significant differences versus the respective osteotomy groups.

### Bacteriological examinations

Spot check sampling and analyses of sterile swab tests from 18 animals at different time points after open osteotomy (*i.e*. at 29, 42 and 84 d) revealed positive results for *S. aureus *in 4 animals. Sample collection for these positive swabs was performed twice around the callus and twice intramedullary. In this context, it should be pointed out, that the animals with positive bacteriological results for *S. aureus *showed no macroscopic signs of purulence and must therefore be deemed to be subclinically infected.

## Discussion

In order to evaluate an applicable *in vivo *model for local therapeutical targeting, we investigated the impaired healing course of a rat tibial osteotomy with intramedullary stabilization compared to a previously described closed fracture model with regular healing.

Surgical procedure of rat tibiae osteotomy as performed in this study or applied in other studies [27,28] generates a unique phenotype of bone defect according to the AO-classification for fractures (42A3). Hence, it provides consistent preconditions for further investigations. In contrast, the use of a fracture device could lead to inconsistent fracture types [22].

Radiological, biomechanical and histological screening during the observation showed a clear course of impaired bone healing. Even after 84 d the osteotomized tibiae were far from the stage of remodeling the original bone structure. At best, defect bridging occurred periosteally but not interfragmentary as seen in the fracture model [24]. Primary periosteal bridging is an integral part of the approximately 5 week bone healing process in rats and has been described as species-specific [29]. On the other hand, a contributory factor may be the use of the intramedullary stabilisation technique.

The development of delayed healing after tibial osteotomy in direct comparison to the closed fracture technique was previously documented in a rabbit model by Park *et al*. [30] using an external fixator for stabilization of the bone defect. These authors showed respective differences two weeks post bone defect, but these had disappeared at later observation time points. In a study with rats Kokubu *et al*. [31] developed a femoral model with 100% nonunion up to 56 d post surgery. However, as an effect of cauterization on each side of the fracture, the radiographical appearance of nonunions was atrophic.

Compared to our previously reported study in rats which underwent closed tibial fracture with the same intramedullary stabilization, clear differences in the biomechanical properties of the osteotomized tibiae could be observed in contrast to the fractured tibia. The osteotomized tibiae showed significant lower values for maximum torque and torsional stiffness after 42 and also 84 days as compared to the fractured tibiae. Even though sample preparation for biomechanical testing at an earlier time point (28 d) was not possible for all osteotomized bone samples, mainly due to the very instable callus, the results were in accordance with those in a study from Shefelbine *et al*. [28] demonstrating also very low biomechanical data 21 d after rat tibial osteotomy.

Although our results can only be interpreted cautiously because of the small sample size investigated, the comparative examination of osteotomized and fractured bones in the present study gave evidence, that the course of delayed healing in osteomized animals is determined already before day 28 after surgery.

Histological investigations comparing the two different techniques even in earlier healing phases, at 10 d post bone defect, also revealed differences: The histomorphometric analyses showed that the amounts of accumulated desmal formed reparative bone tissue and of differentiated cartilaginous tissue were retarded in the region of the osteotomy defect compared to the fracture.

Less deposition of trabecular bone could be found on the anterior tibial side for both groups. One compounding parameter may be the weakly developed soft tissue envelope on the anterior tibial side. Moreover, comparison of the regional accumulation of differentiated cartilage revealed that osteotomized animals accumulate lower amounts of differentiated cartilaginous cells on the anterior side where the surgical approach was located. We therefore conclude that the choice of surgical approach can influence the local formation of reparative cartilaginous tissue.

Furthermore, in the callus of osteotomized rat tibiae we found numerous amounts of inflammatory cells. Additionally the number of vessels in callus was significantly higher than in the callus of fractured tibiae, where the amount decreased between 5 and 10 d post intervention. Comparable results *i.e*. an initial relatively constant number of vessels in the callus of tibiae with induced impaired healing in contrast to a decreased number in the regular healing group were shown in osteotomized sheep [32]. We would interpret these observations in the present study as a prolonged and pronounced inflammatory reaction occurring in all osteotomized rats. In fact, microbiological screening revealed persistent subclinical infection in a subset of the osteotomized animals. This can hardly be avoided given the keeping conditions and activities of the laboratory animals. For a rabbit model, Melcher and colleagues [33] showed even higher infection rates. Therefore, adjuvant systemic antimicrobial prophylaxis should be considered in future animal studies. Thus, tibial osteotomy has a higher chance of infection than closed fracture, something that also occurs in surgical treated closed tibial fractures in humans [2].

What could be a reasonable explanation for the delayed bone healing in all osteotomized animals in this study? Radiologically, we could rule out poor end-to-end contact of osteotomy gap. Another frequently quoted cause could be a high degree of instability in the osteotomized bone, concerning the rotation movement [34]. But all animals were stabilized in the same manner as described for the healing fracture model and breed, weight and sex were analogous. Although this was not examined in any comparative measurements, differences in instability were not obvious in either group. However, variations within minimal interfragmentary movements cannot be ruled out. In fractured animals we observed the deposition of more defect-associated cartilage, pointing to a possible higher degree of instability capable of modulating the healing process [35,36]. The comparative analysis of tibia and fibula in osteomized animals may support this hypothesis as more cartilaginous tissue formation was detected around the fractured and unstabilized fibula than around the stabilized tibial gap in histological bone slices from the same individual. However, the fibula was broken manually and the tibia by osteotomy. Both of these parameters can influence the outcome.

Poor vascularization would be an implausible reason for non-union. The calluses appeared hypertrophical radiologically and well vascularised histologically. Bone necroses at the fragment sides were not observed and severe injuries of the bone's nutrient vessels were not conspicuous during surgery.

Furthermore, proliferating mesenchymal stem cells from bone marrow are though to play a key role in bone repair [37,38]. Primarily used for cooling purposes, open techniques require irrigation and it cannot be ruled out that progenitor cells were washed out. However, haematoma removal within the first hour was shown to be of no relevance for the healing progress [39].

The most plausible explanation for nonunions in this study may be an extended spatio-temporal inflammation and granulation phase, identifiable by dimensions, prolonged inflammatory cell composition and initial vascularization. Local infection as detected in a subset of animals may be a contributory factor but aseptic inflammation leading to nonunion [40] could be assumed to play a main role in our model.

As known from *in vitro *cell culture systems, environmental changes have the potential to influence the differentiation and functioning of cells. Regardless of the importance of inflammation for initializing the healing process, severe soft tissue trauma and the linked excessive release of inflammatory mediators can be discussed as factors to have a negative impact on bone healing [41].

Irrespective of the initial trigger, consideration should be given to whether persistent early inflammation could be a basic principle for nonunions.

## Conclusion

The open tibial osteotomy technique combined with intramedullary stabilization in rats leads to pronounced impairment of bone healing. Prolonged local initial inflammation is suggested as a causative mechanism.

We conclude that the reported osteotomy model is not suitable to provide insight into normal bone healing. Nevertheless, it could be used as a delayed healing model. As known from previous studies, PDLLA coating of the titanium implant has no negative effect on bone healing (21,24). Thus, further investigations with therapeutic application of pharmaceutical agents (*e.g*. growth factors) *via *the PDLLA-coated implant should allow evaluation of the potency of locoregionally administered drugs for nonunion therapy.

## Competing interests

The authors declare that they have no competing interests.

## Authors' contributions

BW, GS, CK designed the experiments. Osteotomy and analyses was performed by CB and CK; Fracture model and analyses was performed by BW and SG. BW, GS and GD supervised the manuscript prepared by CK.

## Pre-publication history

The pre-publication history for this paper can be accessed here:



## References

[B1] Einhorn TA (1995). Enhancement of fracture-healing. J Bone Joint Surg [Am].

[B2] Coles CP, Gross M (2000). Closed tibial shaft fractures: Management and treatment complications. A review of the Prospective Literature. Can J Surg.

[B3] Hadjiargyrou M, Ahrens W, Rubin CT (2000). Temporal expression of the chondrogenic and angiogenic growth factor CYR61 during fracture repair. J Bone Miner Res.

[B4] Auer JA, Goodship A, Arnoczky S, Pearce S, Price J, Claes L, von Rechenberg B, Hofmann-Amtenbrinck M, Schneider E, Müller-Terpitz R, Thiele F, Rippe KP, Grainger DW (2007). Refining animal models in fracture research: seeking consensus in optimising both animal welfare and scientific validity for appropriate biomedical use. BMC Musculoskelet Disord.

[B5] Hietaniemi K, Peltonen J, Paavolainen P (1995). An experimental model for non-union in rats. Injury.

[B6] Petite H, Viateau V, Bensaïd W, Meunier A, de Pollak C, Bourguignon M, Oudina K, Sedel L, Guillemin G (2000). Tissue-engineered bone regeneration. Nat Biotechnol.

[B7] Little DG, McDonald M, Bransford R, Godfrey CB, Amanat N (2005). Manipulation of the anabolic and catabolic responses with OP-1 and zoledronic acid in a rat critical defect model. J Bone Miner Res.

[B8] Markel MD, Bogdanske JJ, Xiang Z, Klohnen A (1995). Atrophic nonunion can be predicted with dual energy x-ray absorptiometry in a canine ostectomy model. J Orthop Res.

[B9] Cruess RL, Dumont J (1975). Fracture healing. Can J Surg.

[B10] Simmons DJ (1985). Fracture healing perspectives. Clin Orthop Relat Res.

[B11] Simon AM, Manigrasso MB, O'Connor JP (2002). Cyclo-oxygenase 2 function is essential for bone fracture healing. J Bone Miner Res.

[B12] Rüter A, Mayr E (1999). Pseudarthrosis. Chirurg.

[B13] Wiss DA, Stetson WB (1996). Treatment Alternatives. J Am Acad Orthop Surg.

[B14] Naimark A, Miller K, Segal D, Kossoff J (1981). Nonunion. Skeletal Radiol.

[B15] Reed AA, Joyner CJ, Brownlow HC, Simpson AH (2002). Human atrophic fracture non-unions are not avascular. J Orthop Res.

[B16] Marsh DR, Li G (1999). The biology of fracture healing: optimising outcome. Br Med Bull.

[B17] Runkel M, Rommens PM (2000). Pseudoarthrosis. Unfallchirurg.

[B18] Hierholzer G, Ludolph E, Alabi ZO (1983). Posttraumatic pseudarthroses–an analysis of development and treatment. Arch Orthop Trauma Surg.

[B19] O'Loughlin PF, Morr S, Bogunovic L, Kim DA, Park B, Lane JM (2008). Selection and development of preclinical models in fracture-healing research. J Bone Joint Surg Am.

[B20] Schmidmaier G, Wildemann B, Stemberger A, Haas NP, Raschke M (2001). Biodegradable poly(D, L-lactide) coating of implants for continuous release of growth factors. J Biomed Mater Res.

[B21] Schmidmaier G, Wildemann B, Bail H, Lucke M, Fuchs T, Stemberger A, Flyvbjerg A, Haas NP, Raschke M (2001). Local application of growth factors (insulin-like growth factor-1 and transforming growth factor-beta1) from a biodegradable poly(D, L-lactide) coating of osteosynthetic implants accelerates fracture healing in rats. Bone.

[B22] Schmidmaier G, Wildemann B, Melis B, Krummrey G, Einhorn TA, Haas NP, Raschke M (2004). Development and characterization of a standard closed tibial fracture model in the rat. European Journal of trauma.

[B23] Greiner SH, Wildemann B, Back DA, Alidoust M, Schwabe P, Haas NP, Schmidmaier G (2008). Local Application of Zoledronic Acid incorporated in a Poly(D, L- 14 Lactide) coated implant accelerates achievement of 15 biomechanical stability in Fracture Healing in Rats. Acta Orthop.

[B24] Schmidmaier G, Wildemann B, Ostapowicz D, Kandziora F, Stange R, Haas NP, Raschke M (2004). Long-term effects of local growth factor (IGF-I and TGF-beta 1) treatment on fracture healing. A safety study for using growth factors. J Orthop Res.

[B25] Vialli M (1951). Osservazioni sull'nso dell'Alcian blue 8 GS netto studio dei mueopolysacchavidi. Boll Soc Ital Bioh sper.

[B26] Movat HZ (1955). Demonstration of all connective tissue elements in a single section; pentachrome stains. AMA Arch Pathol.

[B27] Beck A, Krischak G, Sorg T, Augat P, Farker K, Merkel U, Kinzl L, Claes L (2003). Influence of diclofenac (group of nonsteroidal anti-inflammatory drugs) on fracture healing. Arch Orthop Trauma Surg.

[B28] Shefelbine SJ, Augat P, Claes L, Beck A (2005). Intact fibula improves fracture healing in a rat tibia osteotomy model. J Orthop Res.

[B29] Wray JB, Lynch CJ (1959). The vascular response to fracture of the tibia in the rat. J Bone Joint Surg Am.

[B30] Park SH, O'Connor K, Sung R, McKellop H, Sarmiento A (1999). Comparison of healing process in open osteotomy model and closed fracture model. J Orthop Trauma.

[B31] Kokubu T, Hak DJ, Hazelwood SJ, Hari Reddi A (2003). Development of an atrophic non-union model and comparison to a closed healing fracture in rat femur. J Orthop Res.

[B32] Lienau J, Schell H, Duda GN, Seebeck P, Muchow S, Bail HJ (2005). Initial vascularization and tissue differentiation are influenced by fixation stability. J Orthop Res.

[B33] Melcher GA, Metzdorf A, Schlegel U, Ziegler WJ, Perren SM, Printzen G (1995). Influence of reaming versus nonreaming in intramedullary nailing on local infection rate: experimental investigation in rabbits. J Trauma.

[B34] Mølster AO, Gjerdet NR (1984). Effects of instability on fracture healing in the rat. Acta Orthop Scand.

[B35] Ashhurst DE (1986). The influence of mechanical conditions on the healing of experimental fractures in the rabbit: a microscopical study. Philos Trans R Soc Lond B Biol Sci.

[B36] Epari DR, Schell H, Bail HJ, Duda GN (2006). Instability prolongs the chondral phase during bone healing in sheep. Bone.

[B37] Iwaki A, Jingushi S, Oda Y, Izumi T, Shida JI, Tsuneyoshi M, Sugioka Y (1997). Localization and quantification of proliferating cells during rat fracture repair: detection of proliferating cell nuclear antigen by immunohistochemistry. J Bone Miner Res.

[B38] Bruder SP, Jaiswal N, Ricalton NS, Mosca JD, Kraus KH, Kadiyala S (1998). Mesenchymal stem cells in osteobiology and applied bone regeneration. Clin Orthop Relat Res.

[B39] Grundnes O, Reikerås O (1993). The importance of the hematoma for fracture healing in rats. Acta Orthop Scand.

[B40] Green SA, Moore TA, Spohn PJ (1988). Nonunion of the tibial shaft. Orthopedics.

[B41] Bunn JR, Canning J, Burke G, Mushipe M, Marsh DR, Li G (2004). Production of consistent crush lesions in murine quadriceps muscle–a biomechanical, histomorphological and immunohistochemical study. J Orthop Res.

